# PICU Passport: Pilot study of a handheld resident curriculum

**DOI:** 10.1186/s12909-021-02705-9

**Published:** 2021-05-17

**Authors:** Adrian D. Zurca, Conrad Krawiec, Daniel McKeone, Adil Z. Solaiman, Brandon M. Smith, Gary D. Ceneviva

**Affiliations:** 1 Department of Pediatrics, Penn State Hershey Children’s Hospital, P.O. Box 850, 500 University Drive, Mail Code H085, PA 17033 Hershey, USA; 2 Division of General Academic Pediatrics, Nemours/Alfred I. duPont Hospital for Children, Hershey, USA

## Abstract

**Background:**

To explore the impact of an educational tool designed to streamline resident learning during their pediatric intensive care (PICU) rotations.

**Methods:**

Topics and procedures were chosen for inclusion based on national requirements for pediatric residents. Residents received a PICU Passport at the beginning of their rotations. PICU faculty were provided learning objectives for each topic. Residents and faculty were surveyed before and after starting use of the Passport.

**Results:**

Twenty-two residents pre-Passport and 38 residents post-Passport were compared. Residents were more satisfied with their educational experiences (27 % vs. 79 %; *P* < 0.001), more likely to report faculty targeted teaching towards knowledge gaps (5 % vs. 63 %; *P* < 0.001) and felt more empowered to ask faculty to discuss specific topics (27 % vs. 76 %; *P* = 0.002). The median number of teaching sessions increased from 3 to 10 (Z = 4.2; *P* < 0.001). Most residents (73 %) felt the Passport helped them keep track of their learning and identify gaps in their knowledge.

**Conclusions:**

The PICU Passport helps residents keep track of their learning and identify gaps in their knowledge. Passport use increases resident satisfaction with education during their PICU rotation and empowers residents to ask PICU faculty to address specific knowledge gaps.

**Supplementary Information:**

The online version contains supplementary material available at 10.1186/s12909-021-02705-9.

## Background

The American Board of Pediatrics (ABP) and Accreditation Council for Graduate Medical Education (ACGME) have put forth specific learning expectations for pediatric residents [1, 2], and residency programs are tasked with ensuring residents master the breadth of topics specified by these governing bodies during the course of their residencies. Resident learning occurs mostly through direct patient care during a variety of inpatient and outpatient clinical rotations. Due to seasonal variations in patient care, residents’ experiences during their clinical rotations vary even within the same institution. This may lead to different learning opportunities, with some residents receiving little to no exposure to certain important topics since their learning is largely dependent on which patients happen to present during their rotations. As faculty rotate frequently, it can be challenging to understand individual residents’ learning needs. Similarly, residents themselves may not be aware of their knowledge gaps if they have not been exposed to particular patient populations or clinical scenarios.

As adult learners, residents are more likely to learn a concept if they view it as important to be able to solve an immediate problem, relevant to their development [3], and if they are motivated to learn the material [4]. Checklist-based learning tools have been used with medical students in various medical subspecialties [5–7] and emergency medicine residents [8]. To date, the impact of checklist-based tools on inpatient resident education has not been described. Teaching rounds are currently not well characterized, but studies suggest learner-centered education may be occurring less frequently than would be ideal [9, 10].

Most pediatric residency training programs complement the pediatric intensive care unit (PICU) rotation with a combination of a didactic lecture series, required reading assignments, with about one-third offering a PICU handbook for residents developed by the residency program [11]. It is unknown if these methods assist residents in mastering the required core content specifications or ensure residents are comfortable managing a critically ill child. Furthermore, due to the rigors of the PICU rotation, including patient care and other resident duties, it is unknown if residents routinely complete these assigned educational materials.

To address these gaps, we sought to develop a system that improved and streamlined the resident educational experience in the PICU, while maximizing exposure to topics as prescribed by the ABP [1] and ACGME [2]. This is a pre- and post-intervention pilot study aimed to explore residents’ and faculty experiences with the use of a “PICU Passport,” that provides residents and faculty with specific topics to be covered during residents’ PICU rotations.

We hypothesized that the intervention would (1) help improve residents’ satisfaction with their educational experiences during their rotations; (2) allow residents to be better able to track their learning and feel more empowered to approach faculty members to request specific learning topics be covered; and (3) increase the number of teaching sessions and frequency of procedural exposure reported by residents during their PICU rotations. We also hypothesized that use of the PICU Passport would assist faculty in understanding individual residents’ learning needs.

## Methods

### PICU Passport Development

**Fig. 1:**
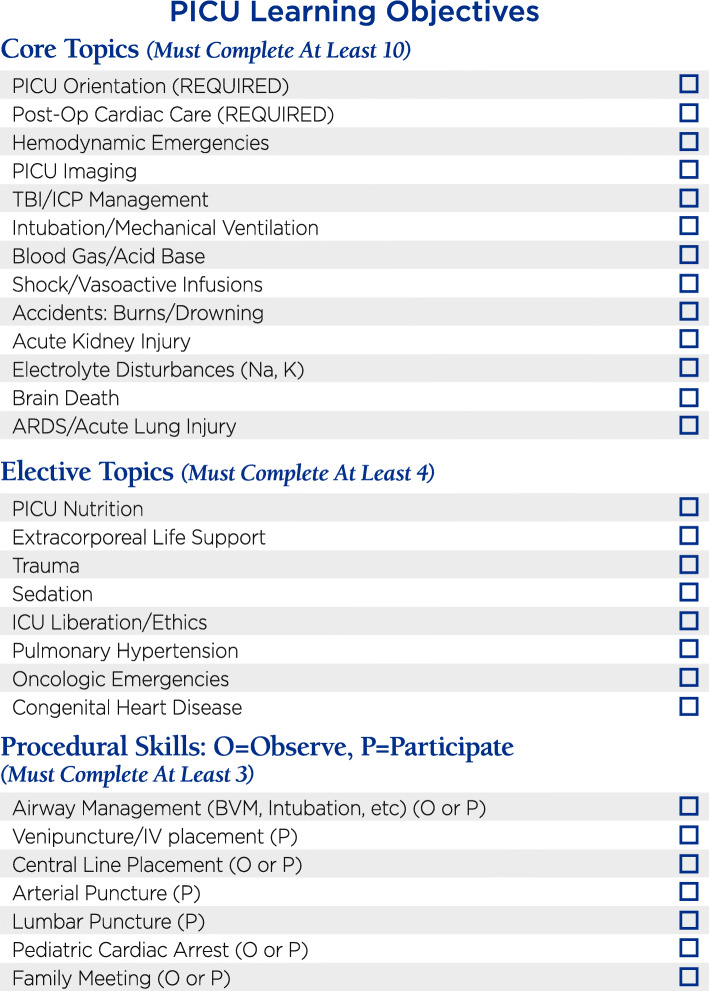
PICU Passport Educational Topics

The authors, including the pediatric residency program director (BS), the pediatric chief residents (DM, AS), and the two co-directors of the PICU resident rotation (CK, AZ) reviewed the content outline published by the ABP for the General Pediatrics certification examination [1] as well as the ACGME program requirements for graduate medical education in pediatrics [2]. After considering resident exposures on other rotations, 21 topics and 7 procedures were chosen as being highest priority for residents at our institution to be exposed to during their PICU rotations. Of the 21 topics, 13 were designated “core” topics and 8 as “elective” (Fig. 1). Subtopics and learning objectives were developed for each of the topics and compiled into a separate document that was distributed to the PICU faculty (Additional file 1).

The procedures chosen included those required by the ACGME as well as others performed almost exclusively in the PICU. Family meetings were included as a procedure to help residents receive exposure to decision-making and end-of-life conversations which are commonly held with families in the PICU. Reference information was also included in the PICU Passport, including phone numbers and medication dosing. The medications and dosing included were compiled by the authors and then distributed for approval to all members of the Division of Pediatric Critical Care Medicine, with adjustments made as necessary.

### Subjects

In our institution, pediatrics residents complete one four-week PICU rotation during each their second and third years; medicine-pediatrics residents (residents completing combined training in internal medicine and pediatrics) complete one four-week PICU rotation during their second year. For the purposes of this study, PICU faculty were defined as any member of the PICU division that shared the responsibility of teaching rotating residents, including PICU attending physicians, fellows, nurse practitiotners, and physician assistants.

### Surveys

De novo survey tools were developed, which included demographics, questions about educational experiences on prior PICU rotations, and residents’ self-reported comfort providing care for critically ill children. The surveys are available as supplemental material. Study data were collected and managed using Research Electronic Data Capture (REDCap) [12].

### Intervention

All pediatric and medicine-pediatric residents at our institution were surveyed before starting to use the PICU Passport. PICU faculty were surveyed at the same time. Residents received the pre-intervention survey in early November, with follow-up invitations sent in December 2015 and January 2016. The follow-up pre-intervention survey invitations were only sent to residents that had (1) not yet completed the pre-intervention survey and (2) not rotated in the PICU after Passport deployment. The PICU Passport was deployed inNovember 2015. Residents who had PICU rotations after the Passport was deployed were no longer eligible to complete the pre-intervention surveys. After starting to use of the PICU Passport, pediatric and medicine-pediatric residents completed surveys at the completion of their PICU rotations. Some residents completed two rotations after starting to use the Passport; however, these residents were only eligible to complete the post-intervention survey after their first post-intervention rotations. The PICU faculty members were surveyed again one year after starting to use the Passport.

All rotating residents received a PICU Passport at the beginning of their four-week PICU rotations. Residents were oriented to the PICU Passport with the expectation that each resident check off 10/13 “core” topics and 4/8 “elective” topics during their rotations. PICU faculty members were provided with learning objectives for each topic (Additional file 1), and were asked to cover requested topics either during rounds, small group discussions, or in one-on-one discussions.

### Statistical analyses

The primary outcomes were originally measured on a 5-point Likert scale. Dichotomous outcome measures were generated by combining the “Agree” and “Strongly Agree” into one category and comparing it to the others. Categorical data was analyzed primarily by Fisher’s exact or Chi square tests. Continuous data was analyzed by Student t-tests for normally distributed variables and Mann-Whitney test for non-normally distributed variables.

## Results

### PICU Residents

Fifty-two of the 56 eligible residents completed the pre-intervention survey from November 2015 to January 2016, for a 93 % response rate. Of these residents, 22 had previously rotated through the PICU and were included for analysis. Over an 18-month study period, 42 residents completed PICU rotations, of which 38 completed the post-intervention survey, for a 90 % response rate. Aggregate survey data was compared between the group of residents who rotated through the PICU before and after starting use of the Passport (Table 1).

**Table 1:** Residents’ Perceptions of PICU Rotation Before and After PICU Passport

	Pre-Intervention *N* = 22 n (%)	Post-Intervention *N* = 38 n (%)	*P*
Level of Training			
- PGY2^a^	3 (13.64)	27 (71.05)	<0.001
- PGY3^a^ (or higher)	19 (86.36)	11 (28.95)	
Feel prepared to recognize a critically ill child	21 (95.45)	36 (94.74)	1
Feel prepared to plan initial management of a critically ill child	18 (81.82)	32 (84.21)	0.46
Comfortable caring for a critically ill child until disposition to a higher level of care	16 (72.73)	22 (57.89)	0.28
Satisfied with educational experience during PICU rotation	6 (27.27)	30 (78.95)	<0.001
Empowered to ask PICU faculty to cover specific topics	6 (27.27)	29 (76.32)	<0.001
PICU faculty targeted teaching towards gaps in knowledge	1 (4.55)	24 (63.16)	<0.0001
Acquired skills and knowledge during PICU rotation important to general pediatrics residency training	17 (77.27)	35 (92.11)	0.13

^a^*PGY* Post-graduate year of training

There was no difference in residents’ perceived abilities to recognize, plan the initial management of or care for a critically ill child (Table 1). Of the 38 residents that used the Passport during their rotations, most felt the PICU Passport helped them identify potential gaps in their knowledge and skills (70 %) and helped them keep track of their learning (65 %). Almost all residents felt that the Passport was easy to use (97 %).

#### Resident satisfaction and educational experiences

Residents using the Passport reported increased satisfaction with their educational experiences, were more likely to state faculty chose to discuss educational topics targeted towards residents’ individual knowledge gaps and that they felt empowered to ask faculty to discuss specific topics. Since the pre-intervention cohort was mostly comprised of residents PGY-3 or greater, the analysis was repeated comparing only the senior residents in both the pre- and post-intervention cohorts (Table 2). The median number of teaching sessions reported during the rotation increased from 3 to 10 (Z = 4.2; *P* < 0.001).

**Table 2:** Third-Year Residents’ Perceptions of PICU Rotation Before and After PICU Passport

	Pre-Intervention *N* = 19 n (%)	Post-Intervention *N* = 11 n (%)	*P*
Feel prepared to recognize a critically ill child	18 (94.74)	11 (100)	1
Feel prepared to plan initial management of a critically ill child	15 (78.95)	11 (100)	0.27
Comfortable caring for a critically ill child until disposition to a higher level of care	13 (68.42)	10 (90.91)	0.22
Satisfied with educational experience during PICU rotation	5 (26.32)	10 (90.91)	0.002
Empowered to ask PICU faculty to cover specific topics	4 (21.05)	10 (90.91)	0.002
PICU faculty targeted teaching towards gaps in knowledge	0 (0)	6 (54.54)	0.005
Acquired skills and knowledge during PICU rotation important to general pediatrics residency training	14 (73.68)	10 (90.91)	0.37

#### Residents’ procedural exposures

There were no significant differences in the number of procedures residents reported (Table 3).

**Table 3:** Residents’ Reported Exposures to Procedures Before and After PICU Passport

Procedure	Pre-Intervention *N* = 22 n (%)	Post-Intervention *N* = 38 n (%)	*P*
Intubation (Observe)	18 (81.81)	30 (78.95)	1
Intubation (Perform)	9 (40.91)	11 (28.95)	0.57
CVC^a^ (Discuss Indications)	10 (45.45)	26 (68.42)	0.1
CVC^a^ (Observe)	17 (77.27)	30 (78.95)	1
CVC^a^ (Attempt)	7 (31.81)	10 (26.32)	0.77
Family Meeting	15 (68.18)	15 (39.47)	0.06
CPR^b^ (Observe)	12 (54.54)	13 (34.21)	0.18
CPR (Perform)	6 (27.27)	10 (26.32)	1
Arterial Puncture	8 (36.36)	22 (57.89)	0.18
Lumbar Puncture	13 (59.09)	27 (71.05)	0.4

^a^*CVC* Central Venous Catheter

^b^*CPR* cardiopulmonary resuscitation

### PICU Faculty

All 16 PICU faculty completed the pre-intervention survey. 10/17 completed the post-intervention survey.

#### Faculty satisfaction with residents’ educational experiences

There was no significant difference pre- vs. post-intervention in PICU faculty’s confidence in residents being able to either recognize or plan the initial management of a critically ill child (Table 4). Post-intervention, PICU faculty were significantly more likely to be satisfied with residents’ educational experiences during their PICU rotations (Table 4).

**Table 4:** PICU Faculty Members’ Perceptions of Residents Before and After PICU Passport

	Pre-Intervention *N* = 16 n (%)	Post-Intervention *N* = 10 n (%)	*P*
Level of Training			
- Attending	9 (56.25)	6 (60.00)	1
- Fellow, NP, or PA^a^	7 (43.75)	4 (40.00)	
Residents able to recognize critically ill child.	11 (68.75)	9 (90.00)	0.35
Residents able to effectively plan the initial management of a critically ill child.	2 (12.50)	3 (30.00)	0.34
Highly satisfied with residents’ educational experiences in PICU.	0 (0)	4 (40.00)	0.01

^a^*NP* Nurse practitioner, *PA* Physician assistant

#### Faculty perspectives on passport and tracking resident learning

Almost all PICU faculty felt the Passport was easy to use (80 %) and helped them decide on teaching topics (80 %). Half of faculty felt the Passport helped them keep track of residents’ learning, whereas only one of the PICU faculty felt they were able to keep track of residents’ learning needs prior to implementing the Passport (*P* = 0.001).

## Discussion

With the PICU Passport we have provided residents and faculty with a handheld educational tool that directly links to an ABP and ACGME-mandated curriculum. The PICU Passport helps ensure residents receive baseline exposure to important topics during their PICU rotations while targeting individual knowledge gaps, and provides a model that can be adapted for used in other resident rotations. By assisting PICU faculty in understanding their learners’ previous educational exposures, empowering residents in taking control of their education, and specifically incorporating patient care-focused teaching during rounds and at the bedside, we have taken steps to improving resident education in the PICU, while ascribing to the andragogy model of adult learning theory [3].

The PICU can be a hectic and volatile learning environment, and PICU faculty rotate service every week and may not have more than one call with a resident. While rich in educational experiences, time for traditional didactic learning can be limited by patient care, intermittent faculty interaction during rounds, and administrative tasks. With resident duty hours restrictions limiting interaction with residents, PICU faculty may struggle to understand what individual residents may have already been exposed to and what their learning needs may be. Novel methods are needed to assist faculty members and learners to efficiently encourage learner-centered education and make the most of limited teaching time. PICU faculty are challenged to efficiently incorporate teaching into rounds [13] and having a better understanding of learners’ needs can help faculty be more efficient in their educational efforts. In this study, PICU faculty indicated the Passport helped guide decisions on teaching topics for residents. Only one faculty member felt that they were able to keep track of resident learning before using the Passport, with half of faculty ultimately indicating that the Passport helped them keep track of resident learning.

Use of the PICU Passport was significantly associated with improved resident satisfaction with their educational experiences during their PICU rotations. Perhaps more importantly, residents reported feeling significantly more empowered to ask for specific gaps in knowledge to be addressed. As adult learners, medical trainees enter educational opportunities with experiences that impact their abilities to learn, and more readily learn things deemed important to help them cope with real-life situations [14]. Thus, by making clinical learning more learner-centered and therefore directly relevant to residents, the PICU Passport can help ensure that residents get the most out of limited faculty teaching time.

The lack of significant improvements in residents’ exposure to procedures in the PICU was disappointing, but not surprising when considering that the ACGME does not require pediatric residents to demonstrate proficiency in many of the procedures usually performed in the PICU [2]. Procedural experiences tends to preferentially be generally given to other learners in the PICU, such as pediatric critical care medicine fellows or nurse practitioners, who are required to learn and use these skills as part of their training and clinical responsibilities. In one multi-center study of pediatric airway management, residents took the first attempt at endotracheal intubation only about one-third of the time [15].

It is also important to note that less than half of residents in our study reported the opportunity to observe or participate in cardiopulmonary resuscitation (CPR) during their PICU rotations. While pediatric cardiac arrests are rare and outpatient outcomes are poor [16], inpatient cardiac arrest outcomes appear better [17]. Given the relative rarity of critically ill children outside of the PICU [18], pediatric residents likely receive most of their exposure to critically ill children requiring resuscitation during their PICU rotations. Unfortunately, there is evidence that most graduating pediatric residents struggle to obtain competence in the medical knowledge, technical and communication skills necessary for the emergent management of a critically ill child [19]. Providing more frequent hands-on opportunities to practice resuscitation skills with simulated patient encounters may help increase residents’ abilities to perform resuscitations [20]. Simulation may be used as a tool to provide formative feedback to pediatric residents regarding their communication and acute resuscitation skills; however, it does require significant time and financial resources [21]. Further, current simulation technology is not yet able to truly replicate a real scenario [22]. Tools such as the PICU Passport can be used to track pediatric residents’ participation in actual resuscitation events, helping ensure no resident graduates residency without having participated in at least one actual pediatric resuscitation. Finally, we were interested to find differences in PICU faculty and residents’ perceptions of residents’ abilities to plan the initial management of a critically ill child. Most residents felt prepared to plan the initial management, while only 30 % of faculty agreed. This discrepancy may indicate either resident overconfidence or faculty underestimating residents’ abilities. Prior studies have indicated that residents overestimate their own abilities when dealing with acute resuscitation situations [23]. In one study of pediatric residents’ proficiency performing neonatal endotracheal intubation, an ACGME-mandated requirement, no residents were found to be competent; however, almost all residents felt highly confident in their endotracheal intubation skills [24]. Future studies should aim to further explore and address residents’ actual abilities to care for critically ill children, including using more objective methods to measure residents’ abilities.

### Limitations

This study was conducted in a single academic center with low baseline satisfaction, limiting its generalizability. While we focused on self-reported educational experiences, after careful consideration we decided not to try to objectively measure impact of the Passport on residents’ acquisition of critical care knowledge due to (1) Lack of publicly available and validated measures of resident’s pediatric critical care knowledge and (2) Concern that developing de novo assessments of knowledge (i.e. pre- and post-rotation test) would have led to issues with establishing validity and reliability of the tests as well as introduced significant bias as faculty may have then specifically “taught to the test.” We were also unable to account for potential confounders, including a potential Hawthorne effect impacting faculty’s teaching effort, thus limiting causation between implementation of the PICU Passport directly and the observed improvements in residents’ educational experiences. Finally, due to residents’ rotation schedules, we were unable compare the pre- and post-intervention experiences of individual residents and had to instead compare aggregate results of residents against a historical cohort.

## Conclusions

Use of a handheld educational tool is associated with increased resident and faculty satisfaction with residents’ educational experiences during an inpatient rotation. The PICU Passport is a low-cost and easy-to-use method of individualizing resident learning, while targeting topics prescribed by governing bodies.

## Supplementary Information

**Additional file 1.** PICU Faculty Guide and Educational Objectives.

**Additional file 2.** Resident and faculty surveys.

## Data Availability

The dataset for the current study is available from the corresponding author on reasonable request.

## Abbreviations

PICU: Pediatric Intensive Care Unit
ABP: American Board of Pediatrics
ACGME: Accreditation Council for Graduate Medical Education
REDCap: Research Electronic Data Capture
PGY: Post-graduate year of training
CVC: Central Venous Catheter
CPR: Cardiopulmonary resuscitation
NP: Nurse practitioner
PA: Physician assistant
