# Supplementary material for: PICU Passport: Pilot study of a handheld resident curriculum
Source: BMC Med Educ. 2021 May 17;21:281. doi: 10.1186/s12909-021-02705-9 (PMC8130359; doi:10.1186/s12909-021-02705-9)
Supplement: Supplementary file 1 — Additional file 1. PICU Faculty Guide and Educational Objectives. [file 12909_2021_2705_MOESM1_ESM.docx]

**Appendix A: PICU Faculty Guide and Educational Objectives**

**GUIDE FOR USE OF THIS DOCUMENT**

Dear Colleagues,

This document was put together as a guide for PICU attendings, NPs, fellows and PAs and is designed to go along with the PICU Passport that residents rotating in the PICU will be receiving. As per the PICU Passport, the topics in this document have been divided into “Core” topics and “Elective” topics. These topics were taken directly from the guidelines published by the American Board of Pediatrics and the Accreditation Council for Graduate Medical Education which essentially mandate what pediatrics residents are expected to know by the end of residency (and what they will be tested on for their board exam). In conjunction with the Pediatric Chief residents, the list was narrowed down to what we consider to be the most high yield topics. By the end of their PICU rotation residents are instructed to ensure that they have been exposed to 10/13 Core topics and 4/8 Elective topics (see below for example of how it is presented in PICU Passport).

| ***CORE TOPICS: (Must Complete At Least 10)*** | |
| --- | --- |
| PICU Orientation **(REQUIRED)** | □ |
| Post-Op Cardiac Care **(REQUIRED)** | □ |
| Hemodynamic Emergencies | □ |
| PICU Imaging | □ |
| TBI/ICP Management | □ |
| Intubation/Mechanical Ventilation | □ |
| Blood Gas/Acid Base | □ |
| Shock/Vasoactive Infusions | □ |
| Accidents: Burns/Drowning | □ |
| Acute Kidney Injury | □ |
| Electrolyte Disturbances (Na, K) | □ |
| Brain Death | □ |
| ARDS/Acute Lung Injury | □ |
| ***ELECTIVE TOPICS: (Must Complete At Least 4)*** | |
| PICU Nutrition | □ |
| Extracorporeal Life Support | □ |
| Trauma | □ |
| Sedation | □ |
| ICU Liberation/Ethics | □ |
| Pulmonary Hypertension | □ |
| Oncologic Emergencies | □ |
| Congenital Heart Disease | □ |
| ***PROCEDURAL SKILLS (O=Observe, P=Participate) Must Complete At Least 3:*** | |
| Airway Management (BVM, Intubation, etc) (O or P) | □ |
| Venipuncture/IV placement (P) | □ |
| Central Line Placement (O or P) | □ |
| Arterial Puncture (P) | □ |
| Lumbar Puncture (P) | □ |
| Pediatric Cardiac Arrest (O or P) | □ |
| Family Meeting (O or P) | □ |

On the following pages, you will find specific subtopics to go along with each of the Core and Elective topics presented on the PICU Passport. These are only meant to be used as a guide – you are not in any way expected to cover each and every detail of each topic (in fact, for some of the topics I think it would be impossible to effectively cover everything in one teaching session). The purpose of this Passport is not for each of these topics to be presented as a formal lecture – in fact, most of these should be covered either as a natural part of rounds or during impromptu small-group teaching sessions done during down-time during the day or night. What is envisioned is that on a regular basis, residents will be asked “What do you have left to complete on your Passport?” The residents are also asked to keep track of their progress and approach faculty with specific topics they haven’t heard about yet. In this way we can hopefully ensure that all residents are getting a basic exposure to most of these topics, but we are also largely putting the residents’ educational experiences in their own hands. Finally, just because a topic is not on the Passport does not mean that it should not be covered – education always works best when it is directly tied to patient care and individual interest!

**CORE TOPICS**

**PICU Orientation**

- PICU Passport
  - Review “Introduction to PICU Passport” PowerPoint
- Site-specific:
  - Resident roles and expectations
    - Timing
    - Notes
    - Orders
- Note: May be fulfilled by written document and/or online module

**Post-op Cardiac Care**

- Know that there are potential complications to cardiac surgery
  - Low cardiac output syndrome (“post-pump slump”)
    - Recognize that longer bypass time is associated with increased post-operative morbidity and mortality
  - Bleeding
    - Consider coagulopathy, thrombocytopenia
  - Pneumo/Hemothorax
  - Pericardial tamponade
  - Arrhythmia
- Arrhythmias
  - Identify the clinical manifestations of common cardiac arrhythmias
  - Recognize the junctional ectopic tachycardia is common after cardiac surgery
  - Understand the clinical significance of a prolonged corrected QT interval
  - Using electrocardiographic patterns, identify:
    - Premature atrial contractions
    - Premature ventricular contractions
    - Supraventricular tachycardia
    - Ventricular tachycardia
  - Understand the treatment of supraventricular tachycardia

**Hemodynamic emergencies (tamponade, hypertensive emergency, tension pneumothorax)**

- Tamponade:
  - Pericarditis
    - Know the etiologies and clinical manifestations of pericarditis
    - Know the laboratory evaluation of pericarditis
    - Know the microbiology of pericarditis
    - Know the pathogenesis of pericarditis
    - Know the treatment of pericarditis, including the importance of surgical drainage
  - Recognize pericardial tamponade: vital sign changes, clinical exam findings
  - Describe the emergent treatment of hemodynamically compromising tamponade
- Hypertensive emergency:
  - Recognize and plan the therapy for a hypertensive emergency
- Tension Pneumothorax
  - Know the signs and symptoms of pneumothorax
  - Know the appropriate therapy for a child with pneumothorax
  - Know that spontaneous pneumothoraces occur and may recur in young asthenic boys
  - Know that asthma may be associated with pneumothorax and/or pneumomediastinum
  - Recognize that pneumothorax may be a complication of resuscitation and mechanical ventilation

**PICU Imaging**

- Pleural fluid
  - Diagnose the presence of pleural disease with an imaging study of the chest
  - Understand the etiologies of pleural fluid accumulations
  - Describe the characteristics of pleural fluid associated with empyema
  - Know the characteristics of pleural fluid due to chylothorax
  - Understand the importance of draining an empyema
- Pneumothorax, pneumomediastinum
  - Know the signs and symptoms of pneumothorax
  - Know the appropriate therapy for a child with pneumothorax
- Lines
  - Recognize appropriate positioning of a CVL on x-ray
  - Learn the indications, contraindications and complications of line placement: arterial, CVL, chest tube

**TBI/ICP Management**

- Pathophysiology
- Know that the outcome of a head injury is related to the duration and degree of coma
- Know that a linear skull fracture in an infant younger than 1 year of age is a sign of possible child abuse
- Know the signs and symptoms of spinal trauma
- Recognize the clinical and imaging features of epidural hematoma
- Recognize the clinical and imaging features of subdural hematoma
- Know that a sign of impending coma is increasing respirations
- Distinguish between tachypnea that is compensatory versus hyperventilation
- Recognize a unilateral dilated pupil as a sign of uncal herniation
- Management
- Know the long-term neurologic and behavioral consequences of head trauma
- Recognize the neuroendocrine complications of a head injury
- Recognize that cerebral edema is a consequence of head trauma
- Understand the clinical course and management of epidural hematoma
- Recognize the association of cervical cord injury with head trauma
- Recognize that intracranial hematomas can occur in the absence of a skull fracture
- Understand the clinical course and management of subdural hematoma
- Know the role of pharmacologic therapy in acute spinal cord or craniocerebral trauma
- Understand the different modalities available for medical management of elevated ICP

**Mechanical Ventilation**

- Know the parameters of respiratory failure
  - Understand indications, contraindications and complications of intubation
  - Know the clinical manifestations of acute hypercapnia: flushing, agitation, confusion, tachycardia, headache
  - Recognize the combination of arterial blood gas values that indicate chronic carbon dioxide retention (increased PCO2, normal pH, increased serum bicarbonate concentration, increased base excess)
  - Know when to intubate and when to provide oxygen therapy in patients with respiratory failure of various etiologies
- Airway and respiratory
  - Choose the correct endotracheal tube size for children of various ages
  - Know the value of PEEP in a patient with pulmonary edema and/or hypoxemic respiratory failure

**Blood Gas/Acid-Base**

- Normal mechanisms and regulation
  - Understand the pulmonary mechanism for regulating acid-base physiology
  - Know how to calculate the anion gap
  - Know how to calculate an osmolar gap
  - Blood gas analysis
  - Recognize the limitations of capillary blood gas testing
  - Recognize arterial blood gas findings in a patient who has acute respiratory failure (or opiate overdose)
- Acidosis
  - Recognize the clinical and laboratory presentation of metabolic acidosis
  - Recognize the serum findings in clinical disturbances of acid-base balance in the simple disorders, evaluating pH, PCO2, and bicarbonate
  - Plan initial therapy for severe acidosis (metabolic)
  - Know the differential diagnosis of acidosis associated with a high anion gap
  - Know the pulmonary compensatory changes seen in primary metabolic acidosis
  - Know the renal compensatory changes seen in primary respiratory acidosis
  - Know which diuretics produce metabolic acidosis
  - Formulate a differential diagnosis of acidosis with a normal anion gap
  - Recognize the association of chloride and acidosis in the differential diagnosis of metabolic acidosis
- Alkalosis
  - Know that chronic volume contraction can lead to alkalosis
  - Know the pulmonary compensatory changes seen in primary metabolic alkalosis
  - Know the renal compensatory changes seen in primary respiratory alkalosis
  - Know which diuretics produce metabolic alkalosis

**Shock/Vasoactive Infusions**

- Recognition of impending systemic failure
  - General (vital sign changes)
    - Understand that a normal blood pressure reading does not preclude shock
    - Understand the importance of prolonged capillary refilling time in a sick patient
    - Know that environmental temperature influences capillary refilling time
- Shock
  - Recognize the clinical signs of shock due to fluid loss
  - Know the type of fluids to be administered in the treatment of shock
  - Recognize that frequent clinical assessment is required in the treatment of shock
  - Recognize that immediate fluid resuscitation of infants in shock may require more than 20 mL/kg of fluid to improve their clinical conditions
  - Plan initial antibiotic therapy in a child with purpura and possible sepsis
- Dehydration
  - Know that hypotension is a very late sign of dehydration
- Cardiac and circulatory (shock)
  - Know the correct method for cardiopulmonary resuscitation in children of all ages
  - Recognize cardiogenic shock
  - Know that an electrocardiogram and echocardiography should be part of the evaluation of a patient with possible cardiogenic shock
  - Choose the correct drug(s) for the initial management of septic versus cardiogenic shock
  - Know the guidelines for the initial therapy of hypovolemic or septic shock
  - Realize the occasional value of a bone marrow needle to administer fluid intraosseously in a patient in shock
- Myocarditis
  - Identify the clinical manifestations of myocarditis
  - Know the laboratory evaluation of myocarditis
  - Know the microbiology of myocarditis

**Accidents: Burns/Drowning**

- Burns
  - Recognize airway injury in a patient with an acute burn
  - Plan fluid management for a patient with a severe burn
  - Recognize nutritional problems in children with burns
- Near-drowning
  - Know how to treat hypothermia associated with a near drowning
  - Recognize cerebral edema in an asphyxiated patient
  - Understand the factors that predict the prognosis in a patient who has had a near-drowning episode
  - Know that a patient with minimal symptoms following a near drowning may later develop symptoms that require hospitalization
  - Know that acute respiratory distress syndrome may result from a near- drowning after a period of initial recovery

**Acute Kidney Failure**

- Acute renal failure
  - Know the changing fluid requirements in patients with severe oliguria
  - Know that coexisting volume depletion should be corrected in patients with acute renal failure
- Acute live failure
  - Identify the signs and symptoms of impending hepatic failure

**Electrolyte disturbances**

- Hyperkalemia
  - Know the emergency treatment of hyperkalemia
  - Recognize that severe cardiac rhythm changes may begin abruptly in patients with hyperkalemia
  - Know the signs of hyperkalemia
  - Plan the treatment for a patient with hyperkalemia
- SIADH
  - Recognize the serum and urine abnormalities in SIADH
  - Recognize the clinical abnormalities associated with SIADH
  - Know the treatment of SIADH
  - Know that plasma volume is increased in SIADH
  - Recognize how to differentiate SIADH from hyponatremic dehydration
  - Recognize disease conditions and medications associated with SIADH
  - Understand the importance of fluid restriction in the management of SIADH
  - Understand that head trauma can lead to diabetes insipidus or SIADH
  - Understand how to differentiate diabetes insipidus from hypernatremic dehydration (ie, urine specific gravity, urine and serum osmolalities)
- Protein
  - Understand that hypoproteinemia causes generalized edema

**Brain Death**

- Recognize the criteria for brain death
- Recognize the role of neurodiagnostic studies in the determination of brain death

**ARDS/ALI**

- Know the clinical manifestations of acute respiratory distress syndrome
- Know the natural history of acute respiratory distress syndrome
- Know the pulmonary sequelae of acute respiratory distress syndrome
- Know that acute respiratory distress syndrome has multiple etiologies
- Know the major causes of death in children with acute respiratory distress syndrome: sepsis, extrapulmonary multiorgan failure, air leaks
- Oximetry
  - Know the correlation between PaO2 and oxyhemoglobin saturation
  - Understand the value and limitations of pulse oximetry in caring for children with acute pulmonary disease
  - Know the common causes of inaccurate measurement of SpO2

**ELECTIVE TOPICS**

**Nutrition**

- Nutritional problems associated with specific diseases, conditions
- Discuss the indications for TPN

**Extracorporeal Life Support**

- Understand the basics of ECMO
  - VA vs VV
  - Indications and Contraindications
  - Use as bridge towards either recovery or transplant

**Trauma**

- Plan the initial evaluation of an accident victim
- Abdominal trauma
  - Recognize an acute "surgical abdomen"
  - Know the role of a CT scan in acute abdominal trauma
  - Plan the initial evaluation of a patient with probable splenic rupture
- Chest trauma
  - Identify the risk factors for and treatment of hemothorax
  - Identify a flail chest
- Non-accidental trauma

**Sedation**

- Pain management
  - Discuss dosing of different opioids and benzodiazepines, as well as major side effects
- Discuss sedative analgesia in the PICU
  - Sedation algorithms

**ICU Liberation/Ethics**

- Critical care, end of life, and limitations on medical intervention
  - Decisions to withdraw/withhold life-sustaining medical intervention
    - Recognize and apply ethical decision-making when caring for critically ill patients
    - Recognize and apply ethical principles when involved in end-of-life care
    - Recognize and apply ethical principles with regard to limitations on medical intervention
  - Decisions to withdraw/withhold artificial hydration/nutrition
    - Recognize and apply ethical principles when involved in decisions to withdraw/withhold artificial hydration/nutrition
  - Cardiopulmonary resuscitation and "do not resuscitate" (DNR) orders
    - Recognize and apply ethical principles involving cardiopulmonary resuscitation and "do not resuscitate" (DNR) orders
  - Futility
    - Recognize and apply ethical principles regarding the issue of medical futility
  - Persistent vegetative state
    - Recognize and apply ethical principles when caring for a patient who is in a persistent vegetative state
  - Palliative care and pain management
    - Recognize and apply ethical principles involving palliative care and pain management
  - Physician-assisted suicide and euthanasia
    - Recognize and apply ethical principles involving physician-assisted suicide and euthanasia

**Pulmonary Hypertension**

- Pulmonary hypertension and cor pulmonale
  - Understand the risks for pulmonary vascular obstructive disease (Eisenmenger) in patients with untreated large left-to-right shunt lesions with pulmonary hypertension (eg, large VSD, AV septal defect, large PDA)
  - Know the situations in which pulmonary hypertension and cor pulmonale may occur
  - Know that oxygenation may decrease during abnormal sleep, which may cause pulmonary hypertension or exacerbate existing cor pulmonale
  - Know that pulmonary hypertension is potentially reversible
  - Discuss the physiologic conditions that can potentially worsen pulmonary hypertension (hypoxemia, acidosis, agitation)
  - Recognize the different treatment modalities available for a patient having an acute pulmonary hypertensive crisis

**Oncologic Emergencies**

- Spinal cord compression
  - Differentiate the clinical manifestations of spinal cord compression (eg, from a tumor) from those of other myelopathies, and evaluate appropriately
  - Recognize the need for immediate evaluation of children complaining of back pain, lower extremity weakness, and/or bowel and bladder dysfunction to evaluate for spinal cord compression
- Mediastinal mass
  - Recognize wheezing, positional dyspnea, and a chest mass as an indication for immediate evaluation and management due to the risk of acute respiratory failure
- Infection and sepsis
  - Identify varicella as a life-threatening illness in a patient receiving chemotherapy, and know that varicella-zoster immune globulin should be given immediately after exposure to varicella
  - Recognize the need for immediate evaluation of a febrile child who is neutropenic as a result of chemotherapy
- Tumor Lysis syndrome
  - Discuss electrolyte abnormalities with tumor lysis syndrome
  - Recognize indications for emergent dialysis

**Congenital Heart Disease (Note: This is a particularly HUGE topic, no need to try to cover all)**

- Know that cardiogenic shock may be the initial finding in a newborn infant with congenital heart disease
  - Recognize the findings of cardiogenic shock in the newborn infant
  - Know the treatment of cardiogenic shock in the newborn infant
  - Know what important lesions are associated with the shock-like presentation in a newborn infant
  - Distinguish between central cyanosis and acrocyanosis
- Diagnosis:
  - Identify the clinical characteristics of a tetralogy spell
  - Know the cardiac causes of cyanosis in the newborn infant
  - Recognize that the absence of improvement in arterial oxygen content with 100% oxygen in comparison with room air is compatible with the diagnosis of cyanotic congenital heart disease
  - Recognize the clinical features of transposition of the great arteries
- Management
  - Know the complications of polycythemia in a patient with cyanotic congenital heart disease
  - Understand the prognosis for a patient with tetralogy of Fallot
  - Understand the prognosis for cognitive development in patients with cyanotic congenital heart disease
  - Know that a relative anemia can be associated with a stroke in a patient with cyanotic congenital heart disease
  - Know the immediate management of a child with a hypoxic episode
  - Understand the role of ductus arteriosus in cyanotic congenital heart disease and the use of prostaglandin E1 in treatment
  - Know the importance of patent ductus arteriosus in the presentation of hypoplastic left heart syndrome and in coarctation of the aorta
- Congestive heart failure
  - Diagnosis
    - Recognize irritability, dyspnea during feeding, and decreased volume with each feeding as symptoms of congestive heart failure in infants
    - Identify early fatigue, exercise intolerance, anorexia, and cough as symptoms of congestive heart failure in older children
    - Recognize the signs and symptoms of congestive heart failure
    - Identify the important physical findings (eg, edema, hepatomegaly, jugular vein distention, cardiomegaly, gallop rhythm) in congestive heart failure in older children
    - Know how an imaging study of the chest may help diagnose congestive heart failure
    - Understand the association between systemic arteriovenous malformation and congestive heart failure in a newborn infant
  - Recognize the common causes of congestive heart failure in infants and children
    - Understand the role of the pulmonary vascular bed in the presentation of congestive heart failure in infants with large volume left-to- right shunts
