# Supplementary material for: PICU Passport: Pilot study of a handheld resident curriculum
Source: BMC Med Educ. 2021 May 17;21:281. doi: 10.1186/s12909-021-02705-9 (PMC8130359; doi:10.1186/s12909-021-02705-9)
Supplement: Supplementary file 2 — Additional file 2. Resident and faculty surveys. [file 12909_2021_2705_MOESM2_ESM.zip › Faculty Post-Passport EvaluationR3.pdf]

PICU Resident Education

We are interested in learning your experiences teaching residents in the PICU, as well as how to improve their educational experiences while rotating in the PICU.

We appreciate you taking the time to fill out this survey. Please do not hesitate to contact Adrian Zurca (azurca@hmc.psu.edu) with any questions or concerns.

Please read the attached Summary Explanation of Research to learn more about this project.

[Attachment: "HRP-585 - HSPO Summary Explanation Research (NoPHI).docx"]

By clicking below you are acknowledging that you have read this document and agree to participate in this research project.

◯ I have read the above document and agree to participate in this project.
◯ I would prefer not to participate in this project.

Please tell us about yourself.

What is your level of training?
- ◯ Attending
- ◯ Fellow, NP or PA

I feel like most of the pediatrics and medicine-pediatrics residents (PGY2 and greater) at my institution are able to recognize a critically ill child.
- ◯ Strongly Disagree
- ◯ Disagree
- ◯ Neither agree nor disagree
- ◯ Agree
- ◯ Strongly Agree

I feel like most of the pediatrics and medicine-pediatrics residents (PGY2 and greater) at my institution are able to effectively plan the initial management of a critically ill child.
- ◯ Strongly Disagree
- ◯ Disagree
- ◯ Neither agree nor disagree
- ◯ Agree
- ◯ Strongly Agree

The PICU Passport helped me keep track of residents' individual learning needs during their PICU rotation.
- ◯ Strongly Disagree
- ◯ Disagree
- ◯ Neither agree nor disagree
- ◯ Agree
- ◯ Strongly Agree

The PICU Passport helped me when deciding which topics to discuss with residents during or after rounds.
- ◯ Strongly Disagree
- ◯ Disagree
- ◯ Neither agree nor disagree
- ◯ Agree
- ◯ Strongly Agree

I found the PICU Passport to be easy to use.
- ◯ Strongly Disagree
- ◯ Disagree
- ◯ Neither agree nor disagree
- ◯ Agree
- ◯ Strongly Agree

I am highly satisfied with residents' educational experiences in the PICU.
- ◯ Strongly Disagree
- ◯ Disagree
- ◯ Neither agree nor disagree
- ◯ Agree
- ◯ Strongly Agree
