# Supplementary material for: PICU Passport: Pilot study of a handheld resident curriculum
Source: BMC Med Educ. 2021 May 17;21:281. doi: 10.1186/s12909-021-02705-9 (PMC8130359; doi:10.1186/s12909-021-02705-9)
Supplement: Supplementary file 2 — Additional file 2. Resident and faculty surveys. [file 12909_2021_2705_MOESM2_ESM.zip › Resident Post-Rotation EvaluationR3.pdf]

PICU Resident Education

We are interested in learning your experiences caring for critically ill children, as well as how to improve on your educational experiences while rotating in the PICU.

We appreciate you taking the time to fill out this survey. Please do not hesitate to contact Adrian Zurca (azurca@hmc.psu.edu) with any questions or concerns.

Please read the attached Summary Explanation of Research to learn more about this project.

[Attachment: "HRP-585 - HSPO Summary Explanation Research (NoPHI).docx"]

By clicking below you are acknowledging that you have read this document and agree to participate in this research project.

- ◯ I have read the above document and agree to participate in this project.
- ◯ I would prefer not to participate in this project.

Please tell us about yourself.

What type of residency are you in?
- ◯ Pediatrics
- ◯ Medicine/Pediatrics
- ◯ Other

Please describe ______________________________

What is your level of training?
- ◯ PGY1
- ◯ PGY2
- ◯ PGY3
- ◯ PGY4
- ◯ Other

Please describe ______________________________

Have you had prior ICU experience outside of the PICU? (Please check all that may apply):
- ☐ Yes, NICU (Neonatal ICU)
- ☐ Yes, MICU (Medical ICU)
- ☐ Yes, other
- ☐ No

Please describe ______________________________

Have you had a prior rotation in the PICU (Pediatric ICU)?
- ☐ Yes, as medical student
- ☐ Yes, as resident in this institution
- ☐ Yes, other
- ☐ No

Please describe ______________________________

What are your plans after completing residency?
- ◯ Primary care (outpatient)
- ◯ Hospitalist (inpatient)
- ◯ Fellowship training
- ◯ Undecided
- ◯ Other

Please describe ______________________________

I feel like my experiences in residency thus far have prepared me to recognize a critically ill child.
- ◯ Strongly Disagree
- ◯ Disagree
- ◯ Neither agree nor disagree
- ◯ Agree
- ◯ Strongly Agree

I feel like my experiences in residency thus far have prepared me to plan the initial management of a critically ill child.
- ◯ Strongly Disagree
- ◯ Disagree
- ◯ Neither agree nor disagree
- ◯ Agree
- ◯ Strongly Agree

I am comfortable caring for a critically ill child until disposition to a higher level of care can be arranged.
- ◯ Strongly Disagree
- ◯ Disagree
- ◯ Neither agree nor disagree
- ◯ Agree
- ◯ Strongly Agree

Please tell us about your experiences in the Penn State Hershey PICU. Please read the following statements and indicate whether or not you agree.

In answering these questions, please consider your OVERALL experiences during your PICU rotation.

Approximately how many times during your rotation did a PICU faculty member (defined here as an attending, NP, PA or fellow) provide you a teaching session? Please consider both one-on-one and small group sessions, either during or after rounds. ______________________________

I am satisfied with my educational experience during my PICU rotation.
- ◯ Strongly Disagree
- ◯ Disagree
- ◯ Neither agree nor disagree
- ◯ Agree
- ◯ Strongly Agree

I felt empowered to ask the PICU faculty to cover specific topics.
- ◯ Strongly Disagree
- ◯ Disagree
- ◯ Neither agree nor disagree
- ◯ Agree
- ◯ Strongly Agree

The PICU faculty targeted teaching towards gaps in my knowledge base.
- ◯ Strongly Disagree
- ◯ Disagree
- ◯ Neither agree nor disagree
- ◯ Agree
- ◯ Strongly Agree

The PICU Passport helped me keep track of my own learning.
- ◯ Strongly Disagree
- ◯ Disagree
- ◯ Neither agree nor disagree
- ◯ Agree
- ◯ Strongly Agree

The PICU Passport helped me identify potential gaps in my knowledge and skills.
- ◯ Strongly Disagree
- ◯ Disagree
- ◯ Neither agree nor disagree
- ◯ Agree
- ◯ Strongly Agree

I found the PICU Passport to be easy to use.
- ◯ Strongly Disagree
- ◯ Disagree
- ◯ Neither agree nor disagree
- ◯ Agree
- ◯ Strongly Agree

I acquired skills and knowledge during my PICU rotation that are important to my general pediatrics residency training
- ◯ Strongly Disagree
- ◯ Disagree
- ◯ Neither agree nor disagree
- ◯ Agree
- ◯ Strongly Agree

During my PICU rotation I had the opportunity to:

	No	Yes
Be present for an intubation	○	○
Attempt an intubation	○	○
Discuss the indications, contraindications and complications of central venous line placement	○	○
Be present for a CVL (central venous line) or arterial line placement	○	○
Attempt a CVL or arterial line placement	○	○
Take part in a family meeting	○	○
Be present for a pediatric cardiac arrest	○	○
Be an active participant in a pediatric cardiac arrest	○	○
Perform an arterial puncture ("art stick")	○	○
Perform a lumbar puncture	○	○
